# Survival extrapolation using the poly-Weibull model

**DOI:** 10.1177/0962280211419645

**Published:** 2015-04

**Authors:** Nikolaos Demiris, David Lunn, Linda D Sharples

**Affiliations:** 1 Agricultural University of Athens, Athens, Greece; 2 Medical Research Council Biostatistics Unit, Cambridge, UK

**Keywords:** Bayesian survival analysis, heart lung transplantation, life years gained, poly-Weibull models, survival extrapolation, WinBUGS

## Abstract

Recent studies of (cost-) effectiveness in cardiothoracic transplantation have required estimation of mean survival over the lifetime of the recipients. In order to calculate mean survival, the complete survivor curve is required but is often not fully observed, so that survival extrapolation is necessary. After transplantation, the hazard function is bathtub-shaped, reflecting latent competing risks which operate additively in overlapping time periods. The poly-Weibull distribution is a flexible parametric model that may be used to extrapolate survival and has a natural competing risks interpretation. In addition, treatment effects and subgroups can be modelled separately for each component of risk. We describe the model and develop inference procedures using freely available software. The methods are applied to two problems from cardiothoracic transplantation.

## 1 Introduction

In recent studies of (cost-)effectiveness in transplantation, the quantity of interest has been mean survival over the lifetime of the recipients. Two examples are: (1) estimation of the difference in mean survival for recipients of a single lung transplant (SLT) compared with a double lung transplant (DLT), and (2) potential mean life years gained (LYG) using a novel device for preservation of donor hearts during transportation to the recipients; the device is expected to incur high costs during the transplant procedure but the full benefit will only be accrued over several years after heart transplantation. Mean survival is estimated as the area under the survivor curve but, in common with many cases, the complete survivor curve is not observed at the time of the study. Thus, we can either estimate the truncated mean survival defined by the period over which the survivor curve is observed, or we can use some form of survival extrapolation. The former will give an accurate estimate of a quantity that is of limited interest, while the second gives a possibly less accurate estimate of mean survival but answers the question of interest. Therefore calculation of the full benefit of new therapies and comparisons of the life expectancy of different subgroups depends on survival extrapolation.

Extrapolation of survivor curves in cost-effectiveness studies is routine but is often completed in *ad hoc* ways, including extrapolation of constant hazard rates, which at the very least ignores the increasing risk due to ageing. In transplantation, hazards have been observed to be bathtub- or U-shaped. Empirical hazards are high in the period immediately after transplantation due to the procedure itself and associated immunological events. This is followed by a steep decrease to a period of relative stability reflected by constant hazards, and thereafter hazards increase due to the ageing of the participants and the chronic complications of transplantation.^1^ In this context, and others, the bathtub-shaped hazard functions reflect latent competing risks that operate simultaneously in an additive manner. They are common to many surgical applications, for example in the study carried out by Blackstone et al.,^2^ the hazard following heart valve replacement operations was decomposed into additive overlapping components reflecting similar latent competing risks.

One further complication in transplantation is that the cause of death is either unknown or its determination is often not straightforward. For example, an episode of acute rejection of the transplanted organ will result in increased immunosuppression therapy, which in turn leaves the recipient at risk of infections and further immunological compromise. If the patient then dies, it is not clear whether the cause of death is acute rejection, infection or primary donor organ failure, and all three may contribute. Therefore cause of death is often considered unknown so that we cannot model the competing risks directly, we are required to infer the contributions of the competing risks from the shape of the hazard function.

The above features suggest that a parametric model like a mixture may be appropriate for this task. Sufficient conditions for the shape of the hazard rate are given by Glaser.^3^ In particular, he shows that a mixture of two Weibull distributions cannot result in bathtub-shaped hazard rate, correcting a contention due to Kao.^4^ In contrast, poly-Weibull models^5,6^ in which the overall hazard is the sum of several independent components appear natural in this context. Poly-Weibull models have been used in the competing risks framework, typically with different objectives to the ones that concern us here. Specifically, we are not interested in marginal (or ‘net’) hazards, cumulative incidence functions or examination of the effect of removing a risk component. Merely, we shall use such models as flexible parametric assumptions that may be reasonable for extrapolating survival while their parameters retain a natural interpretation. Gelber et al.^7^ used a hybrid approach in which a parametric model is fitted to the tail of the survival curve while the ‘early’ part is treated non-parametrically. The proposed model also has close connections to other ‘rich’ parametric models such as fractional polynomial regression^8^ (FPR) and generalised additive models^9^ (GAM). However, each component in our model retains a competing risks interpretation in terms of the survival outcome. This is not typically the case with the hybrid, FPR and GAM models.

In our first example, comparison of single and double lung transplantation as treatment options over the lifetime of the recipients is of primary interest, and can be combined with data on quality of life in a more general analysis. In the second example, the assessment of a novel organ preservation system, the main focus is estimation of mean survival within a cost-effectiveness analysis. In both these contexts, it is attractive to adopt a Bayesian approach since diverse sources of evidence often need to be synethesised in a coherent framework.^10^ In our applications, the clinical context dictates the nature of the components of the hazard function and this information can be incorporated *via* prior distributions in a Bayesian approach. In addition, simulation-based techniques seem especially advantageous as the correlation structures that may be present are propagated through to the evaluation of (non-linear) functionals of the basic model parameters, in this case, the mean survival. The development of the WinBUGS software^11,12^ has made the routine fitting of fairly complicated models possible and as a result of these transplantation studies, the poly-Weibull model has been programmed into WinBUGS and is freely available. The remainder of this article is organised as follows. The modelling framework is described in Section 2 and in Section 3 we describe the details of the inference procedure. Implementation of the methods to answer substantive questions in cardiothoracic transplantation is presented in Section 4, while this article is concluded with some discussion in Section 5. The details of the Markov chain Monte Carlo (MCMC) algorithm can be found in Appendix.

## 2 Poly-Weibull model

Suppose that an individual is subject to *m* independent sources of risk that operate additively. Assume further that the distribution of each of the components may be sufficiently described by a Weibull form with density *f*(*t* ∣ ν, λ) = νλ*t*^ν−1^ exp(−λ*t*^ν^). Then, the observed event time is said to follow a poly-Weibull distribution. The hazard function arises as the sum of the *m* independent Weibull-type hazards:

(2.1) $$h(t)=\sum_{j=1}^{m}\nu j\lambda jt\nu j-1,$$

the survivor function is

(2.2) $$S(t)=\exp(-\int_{0}^{t}h(s)\text{ds})=\exp(-\sum_{j=1}^{m}\lambda jt\nu j)$$

and the density is

(2.3) $$f(t)=h(t)S(t)=\exp(-\sum_{j=1}^{m}\lambda jt\nu j)\sum_{j=1}^{m}\nu j\lambda jt\nu j-1.$$

In theory, identifiability problems (and improper posterior distributions) can be encountered when the shape parameters ν_1_, ν_2_,…, ν_*m*_ are equal. In practice, in the absence of causes of death, identifiability will also be problematic whenever each of the *m* hazard components are not sufficiently distinct given the data at hand. We will further examine this issue in Section 4.

Covariate effects can be modelled *via* the rate parameters λ_1_, λ_2_,…, λ_*m*_ in the usual way

(2.4) log(λj)=β0j+β1jz1+…+βpjzp.

For complete flexibility, covariate effects may also be incorporated onto the shape parameters,

(2.5) log(νj)=α0j+α1jz1+…+αpjzp.

### 2.1 Mean survival and discounted mean survival

In many health economic applications, mean survival is of primary interest,

(2.6) $$E(T)=\int_{0}^{\infty }S(s)\text{d}s.$$

Since future survival is considered less valuable than current survival, it is usual to discount future survival at an annual discount rate *d*. The discounted mean survival is given by

(2.7) $$E(Td)=\sum_{t=0}^{\infty }\int_{t}^{t+1}S(s)\text{ds}(1+d)t.$$

For convenience, this can be approximated using the trapezium rule over some time horizon *h*,

(2.8) $$E(\overset{\wedge }{Td})=0.5\sum_{t=0}^{h}S(t+1)+S(t)(1+d)t,$$

and in most cases, this can be calculated numerically using simulation or other methods.

## 3 Bayesian Inference

### 3.1 Likelihood

We shall be concerned with inference based on observed survival data {*y* = (*t*_*i*_, δ_*i*_), *i* = 1,…, *n*}, where for individual *i*, *t*_*i*_ denotes the time to the event or to censoring and δ_*i*_ indicates whether *i* was censored (δ_*i*_ = 0) or not (δ_*i*_ = 1). When the observations of distinct individuals are conditionally independent, the likelihood function can be written as

(3.1) $$L(\nu ,\lambda |y)=\overset{n}{\underset{i=1}{\Pi }}S(ti)h(ti)\delta i,$$

where *S*(*t*) and *h*(*t*) are defined in (2.2) and (2.1) while ***ν*** and ***λ*** denote the vectors of the shape (***ν***_1_, ***ν***_2_,…, ***ν***_*m*_) and rate (***λ***_1_, ***λ***_2_,…, ***λ***_*m*_) parameters, respectively.

### 3.2 Prior specification and identifiability

The choice of priors should be dictated by subject-matter knowledge. It has been empirically observed that a bathtub shape is the most common shape of the hazard rate following transplantation. This suggests that *m* = 2 (often referred to as the bi-Weibull model)^13^ and *m* = 3 are the most likely candidates. In all the examples, considered values for *m* between 1 and 4 were investigated, with the case *m* = 1 used to confirm the validity of our results when compared to the standard Weibull model.

In the case of no covariate effects, we specify vague, log-normal priors for the rate parameters: log λ_*j*_ ∼ Normal(0, 100^2^), *j* = 1,…, *m*. If covariate effects are included, however, we specify Normal(0, 100^2^) priors for the coefficients of those effects. For the shape parameters, we note that the immediately post-operative hazard must be decreasing, and so ν_1_ ∈ (0, 1) (assuming the first poly-Weibull component is designated as corresponding to immediately post-operative risk). An obvious prior choice is then ν_1_ ∼ Uniform(0, 1). Subsequent components of risk may be either increasing or decreasing, and so for these, we specify log-normal priors with mode 1: $\log\nu j\sim \text{Normal}(0,\sigma_{\nu }^{2})$, *j* = 2,…, *m*. We choose σ_ν_ so that shape parameters greater than 15 have only 1% prior probability: σ_ν_ = log(15)/2.33 = 1.16. Note that this induces greater prior weight to the region where ν_*j*_ > 1 but does not preclude ν_*j*_ < 1 values. We will not usually wish to apply covariate effects to the shape parameters, but in cases where this is desirable, we again specify vague Normal(0, 100^2^) for the coefficients, α_0*j*_, α_1*j*_, etc.

To ensure identifiability of the individual hazard components we impose an ordering constraint on the shape parameters, such that ν_1_ < ν_2_ < ··· < ν_*m*_. This is necessary because, with the same parameters, the individual components have identical likelihood contributions. Hence, if unordered, the parameters might switch roles during the MCMC simulation giving, for each parameter, posterior distributions that are a mixture over the supported values. The constraint is applied by multiplying the joint prior by an indicator function $\gamma =\overset{m-1}{\underset{j=1}{\Pi }}I(\nu j+1-\nu j)$, where *I*(*x*) = 1 if *x* > 0, and *I*(*x*) = 0 otherwise.

In cases where covariate effects are to be included for the shape parameters, then the ordering constraint is somewhat more difficult to apply, except in the absence of covariate effects for the rates, where we would be able to impose λ_1_ < λ_2_ < ⋯ < λ_*m*_ instead, say. When covariate effects are included for both sets of parameters, we impose the constraint that ν_1_ < ν_2_ < ⋯ < ν_*m*_ for all possible combinations of observed covariate values. The indicator γ then becomes $\overset{2}{\underset{k=1}{\Pi }}\overset{m-1}{\underset{j=1}{\Pi }}C\mathrm{kj}$, where *C*_1*j*_ = *I*(min_*l*_{*w*_(*j*+1)*l*_} − min_*l*_{*w*_*jl*_}) and *C*_2*j*_ = *I*(max_*l*_{*w*_(*j*+1)*l*_} − max_*l*_{*w*_*jl*_}). Here, *w*_*jl*_ denotes the *l*th element of the 2^*p*^-dimensional vector *w*_*j*_ = *W* α_*j*_, where *p* is the number of covariates, α_*j*_ = (α_0*j*_, α_1*j*_,…, α_*pj*_)′ and *W* a 2^*p*^ × (*p* + 1) matrix containing ones in column 1, and every possible combination of minimum and maximum observed values for each covariate in columns 2–(*p* + 1), e.g. when *p* = 2:

W=(1minz1minz21minz1maxz21maxz1minz21maxz1maxz2).

Techniques for imposing such constraints in BUGS using ‘auxiliary data’ are illustrated in Appendix. In contrast to our approach, the methods presented by Davison and Louzada-Neto^6^ using Laplace's method are not straightforward to extend when covariates are available and an alternative approach based on Gibbs sampling was developed by Mazucheli et al.^14^ who appear to have overlooked the label switching issue.

### 3.3 Implementation

The details of the MCMC algorithm we used can be found in Appendix, including details of the implementation of the poly-Weibull distribution in the WinBUGS Development Interface.^15^ Evaluating the summaries of interest to health economists, such as the mean survival and the LYG for different covariate levels is straightforward in our simulation-based approach. Specifically, sampling from non-linear functionals of the basic model parameters requires no additional coding. For each model fitted, we calculated the deviance information criterion (DIC) described in Spiegelhalter et al.^16^ However, due to skewness in some of the posterior distributions, which can lead to poor estimates of the ‘effective number of parameters’, we prefer to use the posterior mean deviance, penalised by the actual number of parameters (since the model is non-hierarchical), as our model selection criterion, alongside consideration of coefficients' credible intervals. Plummer^17^ discusses the performance of DIC within the framework of penalised loss functions and cross-validation. On related work, Draper and Krnjajic^18^ discuss the equivalence of minimising the DIC and maximising the log-scoring rule using cross-validation. This connection was first established by Stone^19^ for the Akaike information criterion.

## 4 Application to transplantation data

In Section 1, we briefly described the two studies in cardiothoracic transplantation that motivated this study. The poly-Weibull models were developed to estimate mean survival after transplantation where survival extrapolation was required. The first problem relates to different lung transplant procedures and the second is concerned with the effect on life expectancy of cold ischaemic time (IT) in heart transplantation.

### 4.1 Assessing different transplant types

**Figure 1.**
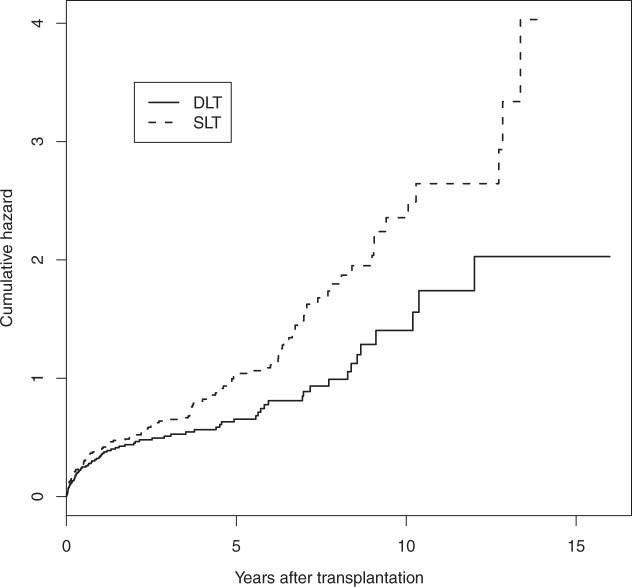
Cumulative hazard estimates for lung transplant recipients.

**Figure 2.**
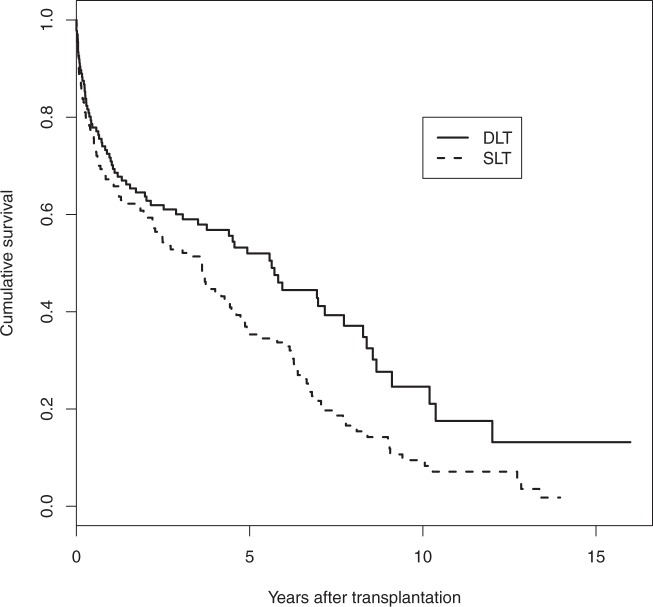
Product-limit survival estimates for lung transplant recipients.

Single lung transplantation (SLT), as opposed to double lung transplantation (DLT), may be used for some patient groups with severe lung disease and it allows two patients to be treated by a single organ donor. In addition, a donor may have only one suitable lung. However, SLT may be considered as a partial treatment and there is some evidence that post-transplant survival may be worse for SLT recipients.^20^ However, whether the effect is mainly associated with poorer operative survival, background survival or chronic complications remains unclear. In addition, the impact on life expectancy for the transplant types is important when deciding whether to accept a SLT when offered. Data from Papworth, one of the UK's specialist cardiothoracic hospitals with a programme of heart and lung transplantation, were used. The dataset consists of 338 patients presenting for lung transplantation mainly due to either chronic obstructive pulmonary disease or pulmonary fibrosis, conditions for which single lung transplantation is a possibility. There were 173 single and 165 double lung transplants out of whom 144 and 79, respectively, died before the end of the study, while the remaining 115 patients were alive at the time of data analysis. Cumulative hazards are plotted in Figure 1, while product-limit survival estimates in Figure 2. It is evident from both figures that there is a high death rate which decreases before slowly increasing in the long term. The single lung patients clearly have worse survival. The cumulative hazard plot indicates that this is most evident in the longer term.

A simple Weibull model (*m* = 1) was not sufficient to describe the data. Within the class of bi-Weibull models, several choices exist for the combination of distinct shape and rate parameters for each risk component and transplant type. In particular, a small number of models are hard to choose between by inspecting the posterior summaries for the different parameters. However, all the ‘highly likely’ models possess a common discernible feature: they suggest only small differences in the early phase but SLT recipients show much greater attrition in the longer term component. This finding is intuitively reasonable and appears clinically sensible. Posterior mean deviance estimates for a range of models are given in Table 1.

**Table 1.** Posterior mean deviance for different models

Model	Mean deviance
Weibull (*m*=1) with covariate on rate	979.1
Poly-Weibull (*m*=2) no covariates	966
Poly-Weibull (*m*=2) covariate on rate parameters only	958.7
Poly-Weibull (*m*=2) covariate on ‘late’ rate parameter only	958.2
Poly-Weibull (*m*=2) covariate on rate and shape parameters	977.3

There is a clear improvement in fit for models with two components compared with the Weibull model, and a clear effect of the covariate SLT. However, beyond that, within the bounds of sampling error, the deviance is the same for all the two-component models investigated. Thus, in the interest of parsimony, the two-component model with SLT having an effect on the later hazards only would seem to be the optimal choice. Parameter estimates for this model are given in Table 2. The SLT group had appreciably higher long-term risks but the 95% credible intervals are wide due to the lack of sufficient data to obtain more precise inference results. This suggests we cannot rule out an effect of SLT on other model parameters, and interpretation of estimates of mean survival should be cautious while product-limit survival estimates are plotted in Figure 2.

**Table 2.** Posterior mean and 95% credible intervals for the different shapes (ν) and log-rate (β) parameters

	Parameter estimate (95% credible interval)
β_01_	−1.12 (−1.32, −0.93)
ν_1_	0.54 (0.45, 0.64)
β_02_	−8.67 (−35.7, −5.46)
β_12_	2.42 (0.68, 29.9)
ν_2_	2.54 (1.71, 3.56)
Mean survival SL	4.96 (4.32, 5.75)
Mean survival DL	8.78 (6.14, 13.7)
Survival difference (DL–SL)	3.83 (1.04, 8.72)

Note: SL, single lung and DL, double lung. Subscript 1 (2) refers to the early (late) risk component.

The model with *m* = 3 was not essentially identifiable as the parameter estimates for the second hazard component are very similar to the estimates of the third component. Thus, considering models with *m* > 2 appears unnecessary in this example.

### 4.2 Cold Ischaemic Time effect

Our second study is concerned with the health economic evaluation of a novel device for storing donated hearts for transplantation. The length of time between excision of a heart from the donor and successful implant into the recipient is called IT. Longer IT has a detrimental effect on survival, particularly in the short term.^21^ In the UK, average IT has been steadily increasing in recent years.^22^ Traditionally, donated organs are packed in ice during transfer from the donor hospital to the transplant centre but the new devices store the hearts in warm, oxygenated donor blood, supplemented with nutrients. It is claimed by the manufacturers that the new machine could effectively reduce the IT to 60 min. However, the machine is likely to be expensive and the potential health gains, evaluated over the lifetime of the patients, will dictate whether the intervention can be termed cost-effective or not. Device regulation is less well developed when compared to drug regulation and an analysis of this kind could help to decide whether a randomised controlled trial of the new device is likely to be a good use of resources.

A preliminary analysis of heart transplant activity from the UK was conducted by Goldsmith et al.^23^ This analysis included 4335 patients of whom 2279 died before the end of the study, with median survival of 10.4 years. The mean IT was 191 min and standard deviation was 59 min. The authors used a Cox^24^ proportional hazard regression model with the hazard ratio for IT piecewise constant, with a single fixed change-point after the initial operative hazards had passed. It showed that short ITs can confer a significant advantage in the early (post-transplant) survival. However, the effect appears smaller in the latter segment of the survival curve and in particular, the IT effect beyond the initial period is not ‘statistically significant’. The length of the initial period was hard to estimate using a change-point model but it appeared that the estimate of the overall LYG (defined as the difference in mean survival) was not sensitive to varying the period between 6 and 12 months. One advantage of the poly-Weibull distribution we shall consider is that a choice of the length of the initial period is unnecessary since the various risk components overlap and there is no assumption of an instantaneous jump in relative hazards. A related issue regarding health economic evaluation, is the sensitivity of the estimated LYG to the piecewise-constant relative hazards assumption. Specifically, assuming different IT effects in the early and late phases can cause large changes in the estimated LYG with severe overestimation bias when a common effect is assumed by the model and extrapolated over the lifetime. Choices of this kind are naturally dealt within the parametric approach we adopt in this article. Finally, and perhaps more importantly, any non- or semi-parametric method necessitates some reliable technique for extrapolating the observed survival. Goldsmith et al.^23^ used the estimated hazard rate in years 13–20 to model crudely the shape of the hazard rate beyond 20 years, for which no data were available. In the model we propose here, this problem is inherently accommodated by the parametric survival distribution.

Several models were considered starting with the simplest model with *m* = 1, i.e. the standard Weibull model. The Weibull model was not flexible enough to describe the bathtub-shaped hazards and gave a poor fit. In transplantation, a model with two hazard components can be thought of as corresponding to the early post-operative risk and the long-term risks of chronic complications from transplantation and the ageing process. The long-term risks could potentially consist of more than one part but since we have no reliable information on the cause of death for these individuals they cannot be delineated and it appears that the bi-Weibull model may represent the best practical choice in this case. We loosely refer to the two risk components as the ‘early’ and ‘late’ hazards. Models with *m* = 3 or *m* = 4 appeared unnecessary both by inspecting the posterior estimates of the model parameters and using the estimated mean deviance. A proportional hazards model for risk component *j* was fitted on the rate parameter by assuming that log(λ_*j*_) = β_0*j*_ + β_1*j*_ × IT. (Models with IT effects also on the shape parameters were considered but gave estimated coefficients, for the effects of IT on shape, that were essentially zero.) The results from the fitted bi-Weibull model can be found in Table 3 (referred to as ‘unconstrained model’). Parameter learning was generally very quick for the early part of the hazard while convergence was slower for the later hazard component. In sensitivity analyses, various parameterisations were attempted, including (λ1=φ2φ1, λ2=1φ1φ2, ν1=ψ1ψ2 and ν_2_ = ψ_1_ψ_2_) as proposed by Louzada-Neto,^13^ with negligible effect. This seems natural since the data are more sparse in the latter part, giving rise to a flatter likelihood. Convergence was obtained with a burn in of at most 10 000 iterations. However, due to high autocorrelation in the simulated chains for some parameters, 500 000 iterations of the Gibbs sampler, in total, were performed in order to obtain accurate inferences. On a 3.06 GHz machine, this took around 22 h for the model presented in Table 3.

**Table 3.** Posterior mean and 95% credible intervals for the different IT effect parameters

	Parameters per hazard component		
	ν_*i*_	β_0*i*_	β_1*i*_ (per hour)
Unconstrained model			
Early part	0.289 (0.273, 0.305)	−1.45 (−1.51, −1.39)	0.226 (0.127, 0.323)
Late part	2.58 (2.36, 2.83)	−7.62 (−8.34, −6.98)	−0.195 (−0.475, 0.0830)
IT effect constrained to be positive			
Early part	0.288 (0.272, 0.304)	−1.44 (−1.50, −1.38)	0.197 (0.10, 0.29)
Late part	2.572 (2.353, 2.817)	−7.55 (−8.24, −6.93)	0.067 (0.002, 0.23)

**Figure 3.**
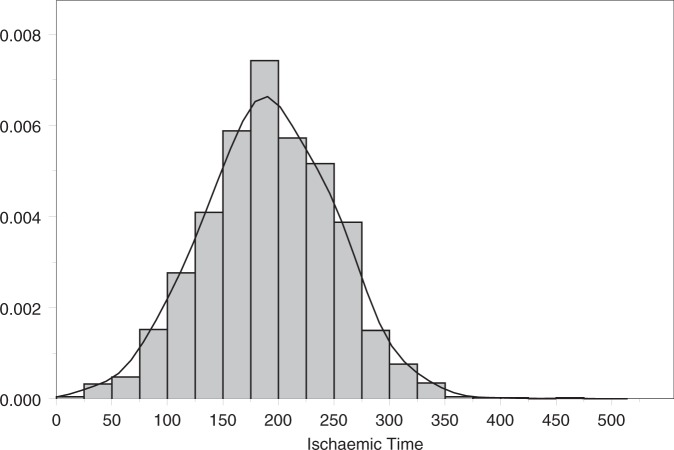
The distribution of the observed ITs.

The estimates we derive are slightly different to the results obtained by Goldsmith et al.^23^ although the findings are not materially different. In particular, the IT effect is strong in the early part of the survival curve but negative, although not ‘statistically significant’, in the latter part. A negative effect of IT is considered implausible by the transplant clinicians, although this was not known when the prior distributions were determined, and has a large effect on the estimates of potential LYG. Mean survival and the LYG for different levels of IT are presented in Table 4. For the model presented in Table 3, i.e. with covariate effects on both rates and no constraints on the second component, a posterior median of −0.901 life years are gained when the IT is reduced from its average of 191 to 60 min. Two alternative assumptions regarding the second component were considered: (1) where the coefficient is constrained to be non-negative (results presented in Table 3); and (2) where the coefficient is fixed at zero. These gave point estimates for LYG of 1.66 and 1.09, respectively. Of course, this assumes that the IT of 60 min can be achieved in practice and that changing IT will result in the predicted change in survival rates, irrespective of other donor and recipient factors. However, it does provide estimates for the potential gain in survival. These results are to be contrasted to the costs of such systems. For example, if health care providers are willing to pay £30 000 per life year gained, the preservation system (including base system, consumables and staffing) must cost no more than £50 000 (1.66 × £30 000) per patient if the constrained analysis is appropriate, or £33 000 (1.09 × £30 000) if the effect is restricted to the early post-operative component. Thus, such machines may not be cost-effective for relatively small IT, but they may prove very cost-effective (as well as clinically effective) in certain circumstances. Long ITs may be less common in Europe (see Figure 3 for the distribution of the observed ITs in the UK) but do arise frequently in countries like Australia and Canada. Further, using such systems may still prove cost-effective in smaller countries when long ITs are anticipated. In summary, our findings suggest that reducing IT could improve average post-transplant survival over the lifetime of the patients by between 1.1 and 1.7 years depending on model assumptions, but the cost-effectiveness of the donor organ maintenance systems will depend on the exact costs and the clinical context in which it is used.

**Table 4.** Posterior summaries for the mean survival, the mean survival if the IT is reduced to 60 min and the corresponding LYG

	Lifetime functions		
	Mean survival	Mean survival at 60 min	LYG
Unconstrained model			
Mean	12.1	11.2	−0.901
95% CI	(11.6, 12.6)	(9.06, 14.0)	(−3.26, 2.04)
IT effect constrained to be positive			
Mean	11.9	13.6	1.66
95% CI	(11.5, 12.4)	(12.6, 15.6)	(0.892, 3.69)
IT effect = 0			
Mean	11.9	13.0	1.09
95% CI	(11.5, 12.4)	(12.4, 13.7)	(0.646, 1.48)

Note: CI, confidence interval.

## 5 Discussion

We have presented methods for survival extrapolation using flexible parametric models that have been previously used within the competing risks framework. The poly-Weibull function has been incorporated into the freely available software, namely WinBUGS, making its implementation straightforward since the details are not more complicated than any other distribution within the package, excepting the requirement for identifiability constraints.

This study was motivated by two problems from the field of cardiothoracic transplantation and these applications demonstrate the simplicity of the approach. The IT example is typical of cost-effectiveness analyses in which there is a high initial cost, but for which the full benefit is only accrued over the lifetime of the patients. Thus, it is important to extrapolate survival in order to estimate the potential benefit and therefore the value of further assessment of the technology. In addition to the poly-Weibull providing a flexible parametric distribution for this purpose, there is no need to make the arbitrary choices surrounding time-varying hazards that were required in a suitable semi-parametric approach. Using this method, we were able to provide some guidance on acceptable costs for the heart preservation system.

There has been recent interest^20^ in the utility of SLT transplants and whether the ability to treat two individuals from the transplant list outperforms the potential LYG from DLT, at least in the cases where both these choices are an option. The comparison between SLT and DLT provides an indication of the difference in life expectancy, which is considerable and, combined with the poorer quality of life associated with SLT, suggests that it should be used selectively. However, credible intervals are wide and this analysis should be revisited in the future. The flexible parametric models of this article could be a significant part of such a study.

In our examples, identifiability issues have been tackled *via* the use of ordering constraints. Occasionally, we may wish to include covariate effects for both the rate and shape parameters simultaneously. In such cases, the proposed constraint is increasingly difficult to satisfy, during the MCMC simulation, as the number of covariates increases. It may be preferable, therefore, to apply some form of post-analysis relabelling algorithm instead, as discussed by Jasra et al.,^25^ to impose the required constraint. Jasra et al.^25^ also discuss more advanced techniques that may be of use in situations where the hazard components are not well separated. Another option may be to incorporate prior information on the model parameters if it is available. Otherwise the model may need to be simplified by combining different sources of hazard. It was not necessary to use strong priors in our studies, and results were robust to a range of prior distributions.

There are alternative approaches for flexible hazard modelling. Various three-parameter distributions have been proposed in the literature.^26,27^ Models of this kind may be appropriate in some cases and they can result in U-shaped hazards. However, some identifiability issues can arise when estimating all three parameters and the choice of the optimal parametrisation is often not straightforward. Also, the interpretation of the model parameters is not always natural in terms of clinical quantities. Finally, incorporating covariates can be complicated in these approaches while both categorical and continuous covariates are simply incorporated within the proposed methods of this article, and retain a natural interpretation.

One potential benefit of using parametric survival curves is that we are able to extrapolate beyond the data and calculate mean survival, a commonly used measure of effect for health economic analyses. This approach allows us to divide hazards into early and late phases, so that later survival extrapolation is less influenced by the early pattern. However, the later phase inevitably contains fewer observations and therefore less information, with poorer mixing of related parameters and typically wide credible intervals, as seen in the IT example. Instabilities may arise in examples with fewer patients with long-term follow-up. Stronger priors may then be required for convergence. Finally, in our experience, posterior distributions can be highly skewed so that DIC estimates can be unreliable. Therefore, unless there is sufficient information extrapolations that rely heavily on the later phase parameter estimates should not be made uncritically.

## Appendix 1

### MCMC algorithm

Implementation of the poly-Weibull model in the freely available WinBUGS software can facilitate its use by researchers in the area. However, model specification using the BUGS language is not straightforward as the likelihood is non-standard. Two basic options are available: (1) the specification of the non-standard likelihood by the use of ‘auxiliary data’ or (2) the implementation of a new distribution in BUGS. The former method introduces an auxiliary observation to ‘replace’ each actual observation (complete or censored) in the data set. These are all assumed to be equal to 1, say, and to have arisen from a Bernoulli distribution with parameter *p*_*i*_. The corresponding likelihood is then Π_*i*_ *p*_*i*_, which provides a mechanism for specifying arbitrary likelihood contributions from each observation, by defining *p*_*i*_ accordingly. However, such an approach can be cumbersome to implement. The second approach of incorporating a new distribution into the BUGS language has several advantages: first, the details of the often complex density are ‘packaged’ within a simple modelling component, making for easier implementation (in particular by other users) and giving a more concise model description, which is less prone to coding errors and easier to read and modify; second, function evaluations implemented in ‘native code’ (Component Pascal) are generally faster; and, finally, the Component Pascal language offers much more flexibility than the BUGS language.

We implement our new distribution by making use of the WinBUGS Development Interface.^15^ With respect to dealing with censoring, which is very common in survival applications, there are two options: to integrate censored observations out of the model or to sample them as part of the MCMC simulation. Although the latter approach is somewhat less efficient, we choose this for several reasons. First, it fits better with the mechanisms already existing within WinBUGS for handling censored data. Second, it helps to maintain the separation of modelling assumptions from data, since the former method would require censoring data to be passed to the poly-Weibull distribution as input. Perhaps most importantly, however, the sampling approach can be used in arbitrary settings/contexts (e.g. prior, likelihood and predictive distribution), without danger of misuse, whereas the integration approach is naturally geared towards a likelihood assumption and could not accommodate, for example, a censored observation with observed descendants in the graph – one can never anticipate all possible uses of a new modelling component. The BUGS syntax for our new distribution is given by T[i] ∼ nd.dpolyweib(m, nu[], lambda[]), where T[i] is the survival time for individual i; m the number of components in the poly-Weibull; and nu[] and lambda[] vectors containing the *m* elements of ν and λ, respectively. In addition, the usual I(.,.) construct can be used for censored observations, as shown in Appendix 2.

As the likelihood is specified in (3.1), WinBUGS only requires random samples from the censored observations. This can be done by solving *P*(*T* ≤ *t*_*_ ∣ *T* > *c*) = *u*, where *t*_*_ denotes the random variate, *c* the point of right censoring and *u* ∼ *U*(0, 1) a sample from the uniform distribution. This translates into solving S(c)-S(t*)S(c)=u or effectively $\sum_{j=1}^{m}\lambda jt_{*}^{\nu j}+\log(S(c)-S(c)u)=0$. It can be shown that a unique solution exists but cannot be obtained analytically unless *m* = 1. We found that a simple Newton–Raphson algorithm was adequate with robust performance for a very wide variety of input combinations. The case of left censoring is treated analogously by solving $\sum_{j=1}^{m}\lambda jt_{*}^{\nu j}+\log(S(b)-(1-S(b))u)=0$, where *b* denotes the left censoring point.

## Appendix 2

### WinBUGS program

Here, we present an example of the programs we used in the IT example. This particular model allows different intercept (beta0) and slope (beta1) for the rate parameters of each of the two hazard components. The ordering constraint for the shape parameters is applied by making use of auxiliary data. We suppose that we have observed a Bernoulli random variable b, equal to 1, and that the associated ‘success probability’ is γ = *I*(ν_2_ − ν_1_). The auxiliary observation b constrains the joint prior *p*(ν_1_, ν_2_) by contributing a likelihood term that only supports ν_1_ < ν_2_.

 for (i in 1:N) {t[i] ∼ nd.dpolyweib(2, nu[], lambda[,i])I(t.cen[i],)}
 for (j in 1:2) {
  for (i in 1:N) {log(lambda[j,i]) <- beta0[j] + beta1[j]*IT[i]}
  beta0[j] ∼ dnorm(0, 0.0001)
  beta1[j] ∼ dnorm(0, 0.0001)
 }
 nu[1] ∼ dunif(0, 1)
 log(nu[2]) <- log.nu2
 log.nu2 ∼ dnorm(0, 0.7432)
 b <- 1
 b ∼ dbern(gamma)
 gamma <- step(nu[2] - nu[1])

Data:

 list(N=4335, t=c(7.292, NA, 0.8295, ...), t.cen=c(0, 10.0246, 0, ...))

If we wish to allow covariate effects for the shape parameters also, then γ is redefined as described towards the end of Section 3.2. For example,

 for (i in 1:N) {t[i] ∼ nd.dpolyweib(2, nu[,i], lambda[,i])I(t.cen[i],)}
 for (j in 1:2) {
  for (i in 1:N) {log(lambda[j,i]) <- beta0[j] + beta1[j]*IT[i]}
  for (i in 1:N) {log(nu[j,i]) <- alpha0[j] + alpha1[j]*IT[i]}
  beta0[j] ∼ dnorm(0, 0.0001)
  beta1[j] ∼ dnorm(0, 0.0001)
  alpha0[j] ∼ dnorm(0, 0.0001)
  alpha1[j] ∼ dnorm(0, 0.0001)
 }
 C11 <- step(min(w[2,1],w[2,2]) - min(w[1,1],w[1,2]))
 C21 <- step(max(w[2,1],w[2,2]) - max(w[1,1],w[1,2]))
 for (j in 1:2) {
  w[j,1] <- alpha0[j] + alpha1[j]*min(IT[])
  w[j,2] <- alpha0[j] + alpha1[j]*max(IT[])
 }
 b <- 1
 b ∼ dbern(gamma)
 gamma <- C11 * C21

For cases in which there are two or more covariates, a more general minmax(.) function, which returns the minimum and maximum values from its vector argument, can be downloaded from the WinBUGS Development web site (http://www.winbugs-development.org.uk/) as a part of the ‘WBDev shared components’.
